# High resolution sequencing of hepatitis C virus reveals limited intra-hepatic compartmentalization in end-stage liver disease

**DOI:** 10.1016/j.jhep.2016.07.048

**Published:** 2017-01

**Authors:** Ditte L. Hedegaard, Damien C. Tully, Ian A. Rowe, Gary M. Reynolds, David J. Bean, Ke Hu, Christopher Davis, Annika Wilhelm, Colin B. Ogilvie, Karen A. Power, Alexander W. Tarr, Deirdre Kelly, Todd M. Allen, Peter Balfe, Jane A. McKeating

**Affiliations:** 1 Centre for Human Virology, University of Birmingham, Birmingham, UK; 2 Ragon Institute of MGH, MIT and Harvard, Cambridge, MA, USA; 3 NIHR Birmingham Liver Biomedical Research Unit, University of Birmingham, UK; 4 School of Life Sciences, Faculty of Medicine and Health Sciences, University of Nottingham, Nottingham, UK; 5 Liver Unit, Birmingham Childrens’ Hospital, Birmingham, UK; 6 Institute for Advanced Study, Technische Universität München, Lichtenbergstrasse 2a, D-85748 Garching, Germany

**Keywords:** Hepatitis C, ESLD, Evolution, Compartmentalization, Innate immunity

## Abstract

**Background & Aims:**

The high replication and mutation rate of hepatitis C virus (HCV) results in a heterogeneous population of viral sequences *in vivo*. HCV replicates in the liver and infected hepatocytes occur as foci surrounded by uninfected cells that may promote compartmentalization of viral variants. Given recent reports showing interferon stimulated gene (ISG) expression in chronic hepatitis C, we hypothesized that local interferon responses may limit HCV replication and evolution.

**Methods:**

To investigate the spatial influence of liver architecture on viral replication we measured HCV RNA and ISG mRNA from each of the 8 Couinaud segments of the liver from 21 patients undergoing liver transplant.

**Results:**

HCV RNA and ISG mRNA levels were comparable across all sites from an individual liver but showed up to 500-fold difference between patients. Importantly, there was no association between ISG and HCV RNA expression across all sites in the liver or plasma. Deep sequencing of HCV RNA isolated from the 8 hepatic sites from two subjects showed a similar distribution of viral quasispecies across the liver and uniform sequence diversity. Single genome amplification of HCV E1E2-envelope clones from 6 selected patients at 2 hepatic sites supported these data and showed no evidence for HCV compartmentalization.

**Conclusions:**

We found no differences between the hepatic and plasma viral quasispecies in all patients sampled. We conclude that in end-stage liver disease HCV RNA levels and the genetic pool of HCV envelope sequences are indistinguishable between distant sites in the liver and plasma, arguing against viral compartmentalization.

**Lay summary:**

HCV is an RNA virus that exists as a quasispecies of closely related genomes that are under continuous selection by host innate and adaptive immune responses and antiviral drug therapy. The primary site of HCV replication is the liver and yet our understanding of the spatial distribution of viral variants within the liver is limited. High resolution sequencing of HCV and monitoring of innate immune responses at multiple sites across the liver identified a uniform pattern of diversity and argues against viral compartmentalization.

## Graphical abstract

**Figure:**
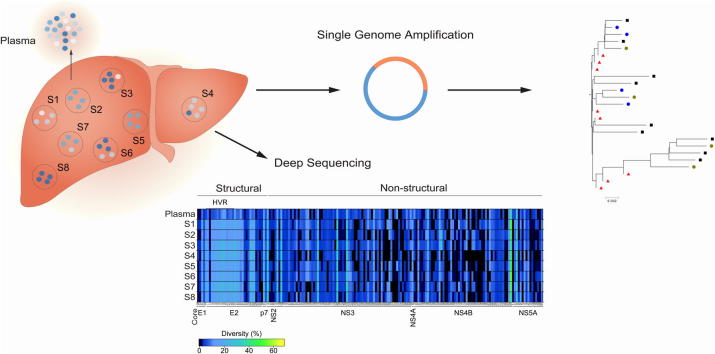

## Introduction

Hepatitis C virus (HCV) is a positive-strand enveloped RNA virus that infects more than 180 million people worldwide, with approximately two-thirds of individuals developing a persistent infection. Chronic hepatitis C is characterized by a long time course, often extending for decades, that can lead to clinical symptoms including fibrosis, cirrhosis and hepatocellular carcinoma (HCC). Infection is characterized by ongoing changes in viral sequences that enable the virus to persist and evade immune surveillance or antiviral therapies [1], [2]. These “swarms” of related viruses have been detected in the liver and peripheral blood or plasma [3], [4], [5], [6], [7].

The major cell type in the liver that supports HCV infection is the hepatocyte and recent studies suggest that 1 to 50% of cells are infected and express viral proteins or RNA [8], [9], [10], [11]. These infected cells occur as clusters surrounded by uninfected hepatocytes, consistent with earlier studies showing HCV dissemination via cell-to-cell contacts [12], [13]. Such isolated foci of infected hepatocytes support a model of intra-hepatic HCV compartmentalization, where viral variants may be localised to discrete regions of the liver. Studies with HIV showed evidence for distinct HIV quasispecies in the white pulp areas of the spleen that contribute to the reservoir of immune escape variants [14], [15], [16]. Our recent study showing that cell-to-cell transmission is the most efficient route for HCV to disseminate *in vitro* [17] supports a model where some viral strains may be retained in the liver and not found in the periphery. Collectively, these studies highlight our limited understanding of HCV spatial genetic diversity within the liver.

Chronic hepatitis C is associated with an active interferon (IFN) response showing high levels of IFN-stimulated gene (ISG) messenger (m)RNAs in the infected liver [18], [19]. HCV has been reported to disrupt host innate immune defenses *in vitro* [reviewed in [20]], however the significance of these evasion strategies in the infected liver are unclear. It is interesting to consider that the clustered pattern of infected cells observed *in vivo* may be explained by local IFN responses limiting viral spread. Indeed, we previously reported that IFNs limit the infectivity of secreted HCV particles [21]. Sheahan and colleagues reported increased ISG mRNA in ‘uninfected’ hepatocytes that were adjacent to HCV infected cells [22], raising questions on the role of this activated bystander response in controlling viral replication and in imposing an immune barrier to HCV sequence diversity.

To investigate whether a spatial interplay exists between host innate immune responses, HCV replication and genetic compartmentalization we measured ISG levels, HCV RNA and the composition and distribution of viral quasispecies in tissue sampled from each of the eight Couinaud segments of the liver and in the plasma of patients undergoing liver transplant.

## Materials and methods

### Clinical samples

Liver tissue and plasma samples were obtained from 21 of 23 patients with chronic HCV infection recruited to the ITX5061 in liver transplant recipients trial [23]. All patients gave specific informed consent and ethical approval was given by the UK National Research Ethics Service (reference 10/H0301/36). A summary of the clinical characteristics and the indications for transplantation, as well as the plasma and hepatic viral loads are detailed in Table 1. Liver tissue from deceased individuals without diagnosed disease were included as normal controls. These livers had been rejected for liver transplantation due to detectable steatosis. On receipt of the explanted liver in the laboratory 250 mg specimens from each of the 8 Couinaud’s segments or a single sample from the control livers were collected for RNA extraction as soon as possible after explant (Table 1). Briefly, snap frozen samples were added to 4 ml of ice-cold RLT buffer (Qiagen, Germany), homogenized using a gentleMACS^TM^ protocol (Miltenyi Biotec, UK), RNA prepared from the homogenate in accordance with the manufacturer’s instructions (RNAeasy Midi kit, Qiagen, Germany) and assessed for possible degradation (Aligent 2200 Tapestation, all RNA integrity number (RIN)>7.5). Viral RNA was purified from 5 ml of plasma (Viral RNA kit, Qiagen, Germany). Plasma samples used in this study were taken within 12 h of the start of the surgery. The amount of HCV in the plasma was quantified in International Units (IU)/ml and converted to HCV copy numbers using the manufacturer’s guidelines (1 IU = 2.7 HCV copies, Cobas Amplicor 2.0 assay, Roche).

**Table 1:** **Individual patient details.**

^1^Two patients were excluded: the liver from patient 10 was damaged by hydatid disease (echinococcosis), patient 12 did not consent to sampling.

### Quantitative Real Time PCR (qRT-PCR)

All cellular mRNA levels were quantified using gene-specific primers together with a glyceraldehyde 3-phosphate dehydrogenase (*GAPDH*) endogenous reference gene (TaqMan® Gene Expression Assays, Life Technologies, UK) in either an ABI 7500 (96 well) or ABI 7900HT (384 well) PCR machine (Applied Biosciences) using a Cells Direct One-Step qRT-PCR kit (Life Technologies, UK). For the detection of HCV a 5′UTR specific primer set was used (Primer Design, UK). All measurements were performed in triplicate in two separate qRT-PCR runs. The quantity of total RNA recovered from each biopsy sample was determined by spectrophotometry, allowing us to adjust the HCV copy numbers according to RNA yield.

### Amplification of HCV structural genes for single genome amplification

HCV cDNAs for SGA were generated using primers specific for either genotype 1a (2616a-1a: GGG ATG CTG CAT TGA GTA, where the name reflects the location (H77 numbering), orientation (sense/antisense) and genotype specificity of the primer) or genotype 3a (3471a-3a: CAA TAG TTC CAA GAA GGC CCC TAG TTT GCT G). cDNA was generated from 5 μg RNA using 0.4 μM antisense primers together with Superscript III reverse transcriptase; cDNA synthesis was performed for 30 min at 55 °C, followed by denaturation at 94 °C for 2 min. Two-step “nested” PCR amplifications were set-up on ice using a Phusion™ High-Fidelity DNA polymerase system (New England Biolabs, GC buffer) in accordance with the manufacturer’s instructions, with the addition of 4% dimethyl sulfoxide (DMSO) to improve yield. The first round primers were: genotype 1 (70s: AGA AAG CGT CTA GCC ATG GCG TTA G and 2616a-1a) or: genotype 3 (70s and 3471a-3a), the PCR amplification conditions were: 30× (94 °C, 15 s; 60 °C, 15 s; 72 °C, 150 s). 2 μl of the completed PCR reaction was added to a PCR mixture containing a sense primer (166s-CAAC: CAA CGT GGT CTG CGG AAC CGG TGA GTA CAC CG) and either a genotype 1a (2582a-1a: TTA CGC CTC CGC TTG GGA TAT GAG TAA CAT CAT) or genotype 3a (3443a-3a: CCC CTA GTT TGC TGG GCG TAT GCT GTG ATC G) antisense primer and re-amplified using the same conditions as in the first PCR. The nested PCR reactions were repeated using a single genome amplification (SGA) end-stage limit dilution procedure to recover single molecules of HCV, cloned (pcDNA™.1D/V5-His-TOPO, Life Technologies) and sequenced, as previously described [24]. All sequences have been deposited in GenBank under accession numbers KX084541–KX084702.

### Deep sequencing of HCV structural and non-structural genes

RT-PCR amplification was performed across the HCV genome using an overlapping amplicon approach to generate near full-length genomes for deep sequencing. For genotype 1a, the first amplicon encompassed the structural proteins from core to NS2 (H77: 279–3542), the second spanned from E2 to NS4B (2290–4774) and the third from NS3 to NS5A (4656–7148). A fourth amplicon covering the remainder of NS5A and the NS5B region failed to amplify. For genotype 3a, the first, second and fourth amplicons were successfully amplified (279–3542, 2486–5776 and 7373–9365 (NS5A to NS5B)) whereas amplicon 3, covering the remainder of NS4B and the 5′half of NS5B, failed amplification. For each amplicon 1–200 ng of hepatic RNA (∼1000 HCV copies) or RNA extracted from 100 μl of plasma (∼10,000 HCV copies) was used as input template. For amplicon 1, the reaction consisted of sense (177s: CCT TGT GGT ACT GCC TGA TAG) and antisense primers (3542a-1a: GGG YAG CAG TTG ACA CRA TCT or 3542a-3a: CTG GGT AGC CGT AGA AAG CAC CT) at 0.4 μM, and a Superscript III RT/Platinum *Taq* Mix in the manufacturers supplied First Strand Buffer (Invitrogen), with the following conditions: cDNA synthesis for 30 min at 55 °C followed by heat denaturation at 94 °C for 2 min, and PCR amplification conditions of 40× (94 °C, 15 s; 58 °C, 30 s; 68 °C 240 s), with a final extension at 68 °C for 10 min. For amplicons 2 and 3 from genotype 1a, this reaction consisted of sense (A2F: AAC GTT GCG ATC TGG AAG AC or A3F: GCT CTC ATG ACC GGC TTT AC) and antisense primers (A2R: GGA AGC GTG GTT GTC TCA AT or A3R: AGA GAT CTC CCG CTC ATC CT) with the same reaction reagents as before but with an adjustment to the PCR amplification conditions, which were 40× (94 °C, 15 s; 55 °C, 30 s; 68 °C 180 s), with a final extension at 68 °C for 5 min. For amplicons 2 and 4 from genotype 3a the primers were : sense (08F: TGG GAT GGG CGY TGA ART GG or 21F: ATG TGT CYG CRG CGC TAG C) and antisense (15R: TAG TTT GGT TGG TCG TCA GG or 25R: AGT AGG AGT AGG CAA AGC AGC) at 0.4 μM, with the same reaction reagents as before but with the following reaction conditions; for amplicon 2: cDNA synthesis for 30 min at 55 °C, heat denaturation at 94 °C for 2 min, then PCR of 40× (94 °C, 15 s; 55 °C, 30 s; 68 °C 180 s), with a final extension at 68 °C for 10 min. For amplicon 4 the PCR conditions were 40× (94 °C, 15 s; 64 °C, 30 s; 68 °C 180 s), with a final extension at 68 °C for 10 min. All PCR products were visualized on 1% agarose gels and purified using the PureLink Quick Gel Extraction Kit (Invitrogen).

For deep sequencing, the PCR amplicons were fragmented and barcoded using NexteraXT DNA Library Prep Kit, as per the manufacturer’s protocol. Samples were pooled and sequenced on an Illumina MiSeq platform, using a 2 × 250 bp V2 reagent kit. Paired-end reads were assembled into a HCV consensus sequence using the VICUNA *de novo* assembler software [25] and finished with V-FAT v1.0. Reads were mapped back to the consensus using Mosaik v2.1.73, and intra-host variants called by V-Phaser v2.0 [26], [27]. All reads have been deposited to the NCBI Sequence Read Archive under the study number SRP065844.

### Assessment of sample diversity

For the deep sequence alignments diversity estimates were calculated as the percentage of reads spanning each coordinate that differed from the patient consensus sequence at a frequency greater than 1% (referred to as percent codon diversity). For example, if 10 of the 27 codons in the hypervariable region (HVR) exceeded this threshold (as seen for patient 1), then the diversity was calculated as 10/27 = 0.3704 or 37.04% [28]. SGA-derived sequences were aligned, genetic distances estimated and phylogenetic trees built using the CLC workbench package (CLC 6.9.1, CLC bio, Denmark).

### Statistical analysis

The viral burden and level of host gene expression was calculated for each replicate. After testing for normality (D’Agostino-Pearson test), all of the measurements made were assessed as logarithmic transforms. Differences between groups were assessed by Mann–Whitney *U* test and correlation coefficients (r^2^) determined by linear regression. *p* values below 0.05 were considered significant. All statistical tests were performed using GraphPad Prism 6.0. pSVR calculations were performed in R using the Random Forest method as previously described [11], [29]. Statistical estimates of compartmentalization were computed using the HyPhy package (http://www.hyphy.org) including; Hudson’s nearest neighbour statistic (S_nn_), which compares the genetic distance between sequences within and between compartments independent of phylogeny (Hudson RR, Genetics 155:2011–14), Wright’s measure of population subdivision (F_st_), and the Slatkin and Madison (SM) statistic.

## Results

### Hepatic interferon stimulated gene expression and HCV replication

**Fig. 1:**
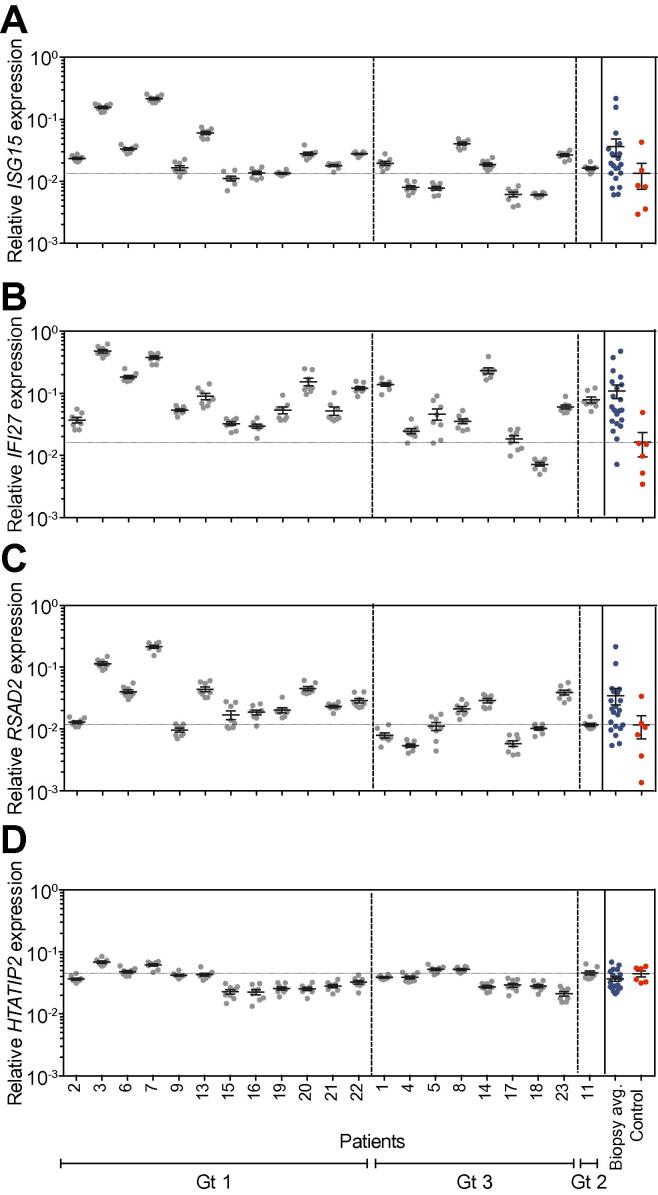
**Interferon stimulated gene expression in the HCV infected liver.** RNA was extracted from biopsies sampled from the 8 segments of the explanted liver of patients diagnosed with chronic hepatitis C (Genotype 1 [Gt1] n = 13; Genotype 3 [Gt3] n = 7 and Genotype 2 [Gt2] n = 1) and from a single biopsy collected from 6 normal controls. The mRNA levels for *ISG15* (A), *IFI27* (B), *RSAD2* (C) and *HTATIP2* (D) were quantified and normalized with an internal *GAPDH* mRNA expression referent. Data are presented for each biopsy sample showing the mean expression levels ± SD and the average values for the total liver denoted in the right-hand panel along with controls (red). The dashed horizontal line denotes the mean expression of normal biopsies.

Biopsies were collected from each of the eight Couinaud segments of the explanted liver obtained from 21 HCV infected subjects undergoing liver transplantation (Table 1). It is well documented that a pre-existing IFN response de-sensitizes the liver to IFN-based therapies. Dill and colleagues screened a large panel of ISGs and discovered three genes; *ISG15*, *IFI27* and *RSAD2* (Viperin) that identified patients with elevated endogenous IFN responses [30]. We therefore measured *ISG15*, *IFI27*, *RSAD2* and *HTATIP2* mRNA (which acts as an internal referent) levels by qRT-PCR in all 8 biopsies sampled from the explant liver. We observed comparable levels of ISG mRNA in all biopsies sampled from a single liver, with limited variability (ranges: *IFI27*, 1.2–5.7-fold (median 2.1); *ISG15*, 0.9–2.2-fold (median 1.6); *RSAD2*, 1.4–3.9-fold (median 1.8)) (Fig. 1A–D). We observed large differences in ISG mRNA levels between subjects: *IFI27*: 68-fold, *ISG15*: 37-fold and *RSAD2*: 43-fold (one-way ANOVA, *p* <0.0001). In contrast *HTATIP2* mRNA levels only varied 3-fold among biopsies sampled from all patients (Fig. 1D). Hepatic biopsies from deceased donors with no diagnosed disease were used as ‘normal’ controls to measure ISG expression. The majority of HCV infected liver explants (19/21) showed increased *IFI27* mRNA levels relative to the controls; whereas approximately half showed elevated *ISG15* (12/21) or *RSAD2* (13/21) expression (Fig. 1A–D). Only 2 patients showed any elevation of *HTATIP2* levels. An algorithm to summarize hepatic ISG expression [11], [29], [31] was calculated and 19 of the 21 explants showed values below 0.5, consistent with a high level of endogenous ISG expression.

Recent studies show the major IFN induced by HCV infected hepatocytes is type III IFNλ [32], [33]. Amplification of *IFNλ* mRNA gave very low signals, with many biopsies producing no PCR product (no amplification in 35 cycles). In those cases, where *IFNλ* mRNA was detectable, the levels were much lower (5000–20,000 fold) than the *IFI27*, *ISG15* and *RSAD2* mRNAs, and similar levels were detected in all 8 biopsies (<10-fold variation) suggesting that, when present, IFNλ is uniformly expressed across the liver (data not shown). Together these data show a remarkably uniform distribution of ISGs in biopsies sampled across multiple segments of the liver, however, we noted a wide range of ISG expression between subjects.

**Fig. 2:**
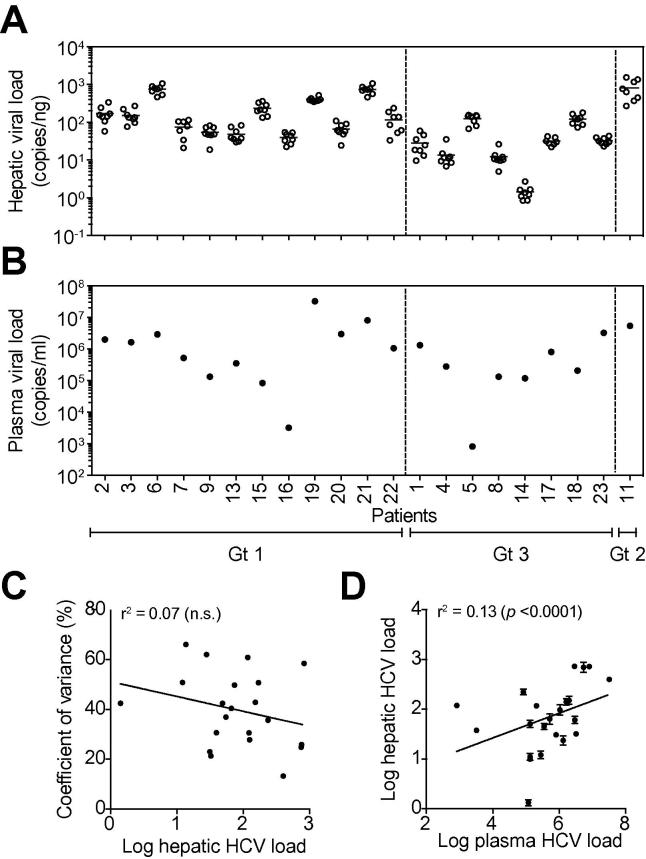
**HCV RNA levels in the liver and plasma at the time of transplant.** The levels of HCV RNA in the hepatic RNA (A) samples described in Fig. 1 along with the plasma-derived RNA (B) were quantified by RT-PCR. Data are presented for each biopsy showing the mean expression HCV RNA/ng total RNA (open circles) and from matched plasma samples showing HCV RNA/ml plasma (closed circles). The mean HCV RNA level ± SD is indicated for each liver. To assess whether the hepatic viral RNA burden (mean HCV RNA of all 8 liver samples) dictates variation across the liver (coefficient of variance – HCV RNA variance/mean HCV RNA) (C) or plasma RNA levels (D) we measured the correlation between these parameters.

In parallel with monitoring ISG mRNA levels in the liver biopsies we measured HCV RNA. The viral RNA burden in the 8 biopsies from a single explant varied from 1.47–6.17-fold (median 3.19), with 7 of the 21 livers showing higher than 5-fold variation across all sites sampled (Fig. 2A). In contrast, the viral RNA burden varied by over 500-fold between patients, from 1.4 (±0.6) to 228 (±48.1) HCV copies/ng RNA. HCV RNA levels were independent of the infecting viral genotype or clinical diagnosis (Table 1). We saw no evidence for any association between intra-patient variance and HCV RNA burden (r^2^ = 0.07, n.s., Fig. 2C). The levels of HCV RNA detected in the plasma at the time of the transplant varied by over 10,000-fold between patients, ranging from 8.2 × 10^2^ to 3.7 × 10^7^ genomes/ml (Fig. 2B). These large variations in plasma and liver HCV RNA led us to assess the correlation between the viral burden in the two compartments. A low but significant correlation was observed (r^2^ = 0.13, *p* <0.0001), suggesting that plasma load is a relatively poor predictor of hepatic burden (Fig. 2D).

There was no correlation between the intra-hepatic ISG mRNA and HCV RNA in the same biopsy or plasma load (*IFI27*: Pearson’s r^2^ = 0.007 (*p* *=* 0.72), *ISG15*: r^2^ = 0.016 (*p* *=* 0.59), *RSAD2*: r^2^ = 0.007 (*p* *=* 0.73)). A combined multivariate analysis of ISG mRNA levels against viral burden was similarly non-significant (r^2^ = 0.039, F-ratio = 0.25, *p* *=* 0.86). In summary, we found no evidence for hot spots of viral replication, with comparable HCV RNA loads detected across all segments in 21 subjects that was independent of ISG expression.

### Hepatic HCV quasispecies

Recent advances with deep sequencing technologies have made it possible to investigate the viral quasispecies in unprecedented detail, allowing us to perform a comprehensive assessment of the distribution and evolution of viral sequences across the liver. From our earlier studies measuring hepatic ISG and HCV RNA levels, we selected patients 1 (genotype 3) and 2 (genotype 1a) as representative cases of the two major infecting genotypes in our cohort. The genomes were sequenced for patient 1 (H77 position 279–5776 and 7373–9365) and patient 2 (279–7148) using deep sequencing (Illumina MiSeq). The mean sequence coverage at each nucleotide position was 5894 ± 1511 (patient 1) and 7465 ± 2399 (patient 2), allowing us to detect variants in the viral population at a frequency of <0.1% [26].

**Fig. 3:**
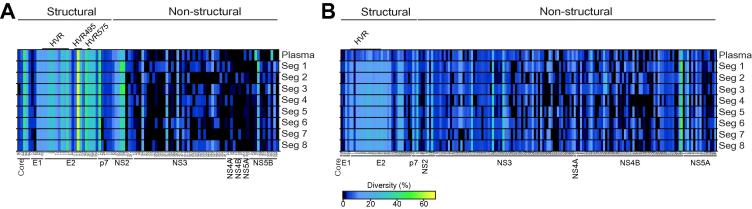
**Deep sequencing of HCV hepatic and plasma quasispecies.** Linear heat map representation of amino acid diversity within the structural and non-structural proteins for the plasma and for the 8 segment biopsies from patient 1 (A) and patient 2 (B). Each square represents a polymorphic codon, coloured to reflect the percentage of reads that exhibit diversity. The diversity scale is colour coded with highly conserved residues in black. Low frequency polymorphisms (<10%) are in dark blue, intermediate levels of polymorphism shade from blue (10%) to green (45%), with the most highly variant residues (45–60%) shaded from green to orange. Only codons exhibiting diversity greater than 1% are shown.

The genetic diversity among all hepatic sites in both patients revealed a striking similarity across the structural and non-structural regions. That is, not only were the same codons variable, the total diversity was similar in all samples (Fig. 3 and Table 3). For instance, within patient 1, there were two residues (L144 and V162; H77 numbering) within the core protein that were polymorphic throughout all samples. The first position was highly diverse with alanine to valine substitutions occurring at frequencies of 35% (segment 8) to 43% (segment 4) (Fig. 3A and Table 3A). At the second site an isoleucine to valine polymorphism was observed, ranging in frequency from 18% (segment 8) to 25% (segment 3). Although a few sites differed between hepatic segments generally all of these sites were low frequency variants at below 5%. Similar data were obtained for patient 2, with the same polymorphisms found across all 8 hepatic segments (Fig. 3B and Table 3B) and only subtle differences observed with low frequency variants. However, one unique polymorphism (A573, NS3) was found to be restricted to a single hepatic segment (segment 5) at a frequency >10% (Fig. 3B). As expected the majority of polymorphic sites were located in the first hypervariable region of E2 (HVR1) (Fig. 3 and Table 3). In patient 1, we noted that the level of diversity in the HVR region was 5 times higher than in the rest of E2, while in patient 2 this diversity was 6 times greater. Although unique low frequency variants were detected in the liver samples, no specific variants were enriched in any particular sample and there was no evidence for signature sequences associated with a hepatic location (Fig. 3 and Table 3).

To further investigate the HCV quasispecies a subset of 6 patients (3 genotype 1a, 3 genotype 3) was selected for SGA by limit dilution PCR. The same hepatic RNA employed for deep sequencing was used as the template for a nested PCR and SGA. A total of 118 SGA-derived HCV E1E2 clones were obtained and sequenced, with an average of 20 clones per patient. Surprisingly we found no evidence for elevated polymorphism in the regions of genotype 3a E2 region previously identified as HVR2 and HVR3 [34], [35], or in HVR495 and HVR575 [36].

The SGA clones allowed us to assess selection pressures by comparing the rate of substitutions at non-synonymous sites (dN) to the rate of substitutions at synonymous sites (dS) across the E1E2 region. The majority of substitutions were synonymous, with observed dN/dS ratios between 0.07 and 0.65 (Table 2). As expected, the majority of dN changes localised to the HVR1, leading to the highest dN/dS ratios in this region (Table 2). Elevated frequencies of dN genetic changes have previously been associated with the innate and adaptive immune response [37]. We saw no correlation between ISG mRNA levels and the dN/dS ratio for E1E2 or HVR1 (E1E2 r^2^ = 0.33 ± 0.027 *p* *=* 0.59, HVR1 r^2^ = 0.05 ± 0.023 *p* *=* 0.25). Taken together, deep sequencing and SGA show similar patterns of viral genetic variation across the liver that are independent of ISG expression during end-stage liver disease (ESLD).

**Table 2:** **HCV Sequence information.**

^1^Mean number of polymorphisms per consensus sequence (Standard Deviation); ^2^Total number of polymorphic sites within the sequence alignment; ^3^Mean *p* value and inferred migration events between liver biopsies and between liver and plasma; ^4^dN/dS-ratio were calculated from the average dN and dS of all pairwise comparisons between sequences; ^5^Patient 5 did not have any amplifiable HCV in the plasma; ^6^No synonymous changes were detected among the plasma derived HVR1 sequences in patient 2. The dN value for this patient was 0.05.

### Plasma HCV quasispecies

Given the relatively poor correlation between hepatic and plasma HCV RNA levels, we were interested to determine whether all members of the hepatic quasispecies were represented in the plasma. Deep sequencing of plasma HCV RNA from patients 1 and 2 showed a similar pattern of diversity to the hepatic segments (Fig. 3 and Table 3). For example, the two variable residues (core L144 and V162) noted in the liver segments from patient 1 were present in the plasma at similar frequencies (L144: 43% *vs.* 40% ± 2.7; V162: 20% *vs.* 22% ± 2.2). Overall, the levels of codon diversity in plasma viral RNA were similar to those measured in the liver (core: 2.62% and 2.55% ± 0.19; E1: 5.73% and 5.14% ± 0.86; E2: 6.23% and 5.86% ± 0.20; p7: 14.28% and 11.11% ± 1.90; NS2: 4.15% and 4.09% ± 0.83; NS3: 3.01% and 3.70% ± 0.86; NS4A: 0% and 2.32% ± 1.64; NS4B: 2.30% and 1.44% ± 1.34; NS5A: 2.44% and 3.05% ± 3.63; NS5B: 0.87% and 1.82 % ± 1.19, respectively). Within NS5B, two threonine codons (T66 and T340) differed between the plasma and the hepatic segments. At position 66 the plasma harbours a dominant alanine codon at this position (61%) with threonine as the minor variant (39%). In contrast the hepatic segments contain a threonine codon (95–99%) with the alanine only rarely seen, at a maximum frequency of 3.8%. Similarly, at position 340 in the plasma, in addition to the dominant threonine codon, a methionine variant was observed at a frequency of 33%. This variant was only seen in 6 of 8 hepatic segments and at a maximum frequency of 2%. For patient 2 concordant patterns of diversity were observed in the plasma and hepatic segments, with similar levels of codon diversity in plasma and liver samples (core: 1.05% and 0.72% ± 0.39; E1: 1.56% and 2.02% ± 0.18; E2: 9.09% and 8.81% ± 0.66; p7: 3.17% and 4.37% ± 1.12; NS2: 7.37% and 6.86% ± 1.51; NS3: 11.09% and 10.76% ± 1.32; NS4A: 3.70% and 1.85% ± 0; NS4B: 14.94% and 15.33% ± 2.61; NS5A: 6.29% and 3.98% ± 0.97, respectively). In this patient one mutation within NS3 (T563) was not detected in plasma but was found in hepatic segments 1,7 and 8 at a frequency of 11–13% (Fig. 3B).

**Table 3:** **HVR polymorphism.** (A) Patient 1 HVR1 polymorphisms detected by Deep Sequencing. (B) Patient 2 HVR1 polymorphisms detected by deep sequencing.




^1^Frequency of sequence (number of reads in brackets); ^2^the difference between the column total and 100% reflects the presence of sequences at a frequency of <1%, 1409 of the 1538 HVR1 sequences (91.6%) detected in this patient are shown here; ^3^the difference between the column total and 100% reflects the presence of sequences at a frequency of <1%, 4385 of the 4683 HVR1 sequences (93.6%) detected in this patient are shown here.

Within the HVR1 we found an average of 9.7 (±0.9) amino acid polymorphisms for patient 1 and 14.7 (±0.7) for patient 2 (considering only those sites with >1% prevalence) (Fig. 3A and Table 3A). The heat maps reflect a diverse quasispecies within the plasma from patient 1, with numerous polymorphisms within HVR1 (Fig. 3 and Table 3). The two viral variants present at greater than 10% frequency were also most prevalent in the liver (Fig. 3 and Table 3). All plasma variants were observed in the hepatic samples, including minority variants. A more homogeneous population was observed in patient 2, with only one major variant present, constituting 73.4% of the plasma sequences and ranging in abundance from 66.3% to 73.5% in the liver. Several minor variants (<5%) were distributed throughout all sites sampled with all variants shared with the plasma at some frequency (Fig. 3B and Table 3B).

The Shannon entropy algorithm can be used to quantify the relative frequency of the variants within a sample. We obtained values of 0.028 (plasma) and 0.021 ± 0.007 (hepatic) for patient 1. Similar values were calculated for patient 2 of 0.017 (plasma) and 0.032 ± 0.005 (hepatic) (Table 2), demonstrating similar levels of polymorphism at all sites. Quantifying the selection pressures acting on each sample revealed a high proportion of synonymous substitutions across both structural and non-structural regions and elevated rates of dN changes within the HVR. This pattern was independent of the site of origin, indicating that both plasma and hepatic quasispecies were evolving in a similar fashion (data not shown). These observations confirm those obtained by SGA sequencing and show concordant results independent of sequencing technology. The high dN substitution rate implies that, even in ESLD, some viral variants may be the result of selective host immune pressure.

### Phylogenetic analysis of HCV compartmentalization

**Fig. 4:**
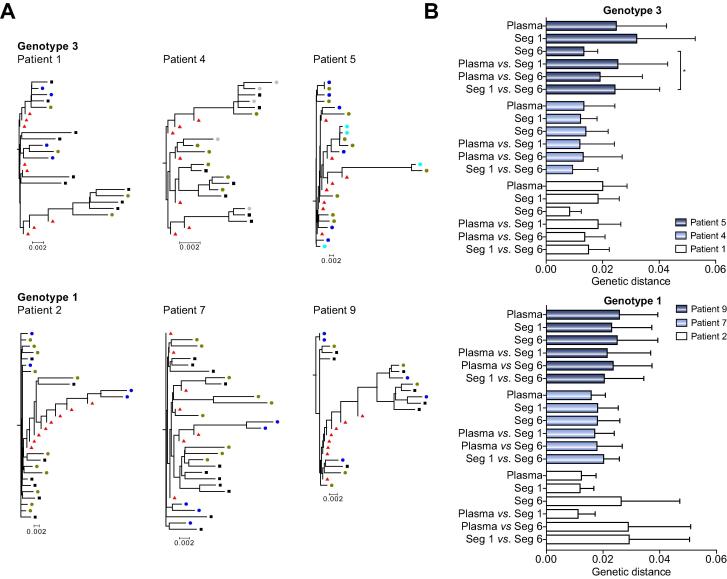
**Phylogenetic relationships between HCV sequences in liver and plasma.** The relationships between HCV E1E2 SGA sequences derived from selected hepatic segments and plasma were assessed by neighbour joining analysis. There was minimal evidence for clustering of SGA-derived sequences originating from the same hepatic segment. The upper phylogenetic trees denote sequences from patients 1, 4 and 5 (genotype 3) while the lower trees represent patients 2, 7 and 9 (genotype 1a). In each tree black squares denote SGA-plasma sequences, whereas coloured circles denote SGA-hepatic sequences derived from segment 1 (green), 3 (cyan), 6 (blue) or 7 (grey). Red triangles represent consensus nucleotide sequences obtained from each of the eight hepatic segments (A). Quantification of mean genetic distance between SGA-derived sequences from identical or different hepatic sites and plasma (B).

Our complete data set of HCV sequences from the liver and plasma allows us to rigorously assess genetic compartmentalization. A phylogenetic comparison by the neighbour joining method of consensus or SGA-derived nucleotide sequences showed no evidence for biopsy-specific sequences (Fig. 4A). Although the sequences obtained from the livers of patients 2, 4 and 5 showed a few pairings, where two SGA sequences from the same hepatic segment grouped together, these clusters included sequences isolated from other sites within the liver. SGA sequences from different liver biopsies were dispersed across their phylogenetic trees, with no site-specific clustering. In patient 7 there was weak evidence for some clustering, with SGA sequences from segments 1 and 6 grouping together and two SGA sequences from segment 1 grouping with their cognate consensus sequence. This was supported statistically by the tree-based SM test (*p* *=* 0.02, inferred migration events: 2). There was no evidence of population structure for the other five patients (mean *p* *=* 0.67, ±0.38, mean inferred migration events: 2.72, ±1.49) (Table 2). When we repeated this analysis using the deep sequencing data from patients 1 and 2, we found no evidence for intra-hepatic compartmentalization (*p* >0.05).

Analysis of sequences from patient plasma showed limited evidence of compartmentalization, with similar genetic distances among the plasma- and liver-derived sequences making it impossible to map the source of the plasma sequences to any specific location in the liver (Table 2;Fig. 4B). Examination of the liver as a single entity also showed no evidence for HCV compartmentalization between plasma using either the SM test (patient 1, *p* *=* 1; patient 2, *p* *=* 1) or two other alternative methods for assessing population structure; Wright’s measure of population subdivision (F_st_) and Hudson’s Nearest Neighbour statistic (S_nn_), (patient 1, F_st_ *p* *=* 0.74, S_nn_ *p* *=* 0.79; patient 2, F_st_ *p* *=* 0.96, S_nn_ *p* *=* 0.42). Overall these data confirm the observations made using the SGA sequences and show that even deep sequencing of the plasma and liver segments failed to detect any major differences between the viral quasispecies at different sites. Furthermore, the simultaneous presence of identical HCV quasispecies variants in the different segments of the liver supports our conclusion that intra-hepatic compartmentalization is not a hallmark of ESLD.

## Discussion

The goal of this study was to assess the spatial relationship between innate immune responses, HCV RNA burden and viral quasispecies diversity across the eight Couinaud segments of the liver from patients undergoing liver transplant. This collection of clinical material provides a novel insight into the relationship between hepatic ISG expression and the viral quasispecies in the liver and periphery.

We used the 4-gene IFN classifier developed by Dill and colleagues [30] to assess ISG expression in the explanted liver and found a remarkably consistent level of expression, regardless of the site of sampling. In contrast, we observed variable hepatic ISG response between patients, irrespective of their diagnosis of cirrhosis or HCC. Importantly, the ISG response across the liver showed no correlation with HCV RNA burden in subjects with ESLD. Using needle biopsies collected in earlier stages of disease, other groups have also failed to observe an association between hepatic ISG expression and HCV RNA levels [9], [30], [38], [39]. We conclude that in chronic late stage hepatitis C innate IFN signaling has limited impact on HCV replication or HCV genetic diversity.

The levels of HCV RNA in all eight biopsies sampled from a single liver were surprisingly similar, suggesting that the liver is uniformly infected during ESLD. Previous studies have shown comparable levels of HCV RNA in biopsies sampled from up to three different regions of a liver [40], [41], [42], [43]. Several studies have suggested that the frequency of HCV infected hepatocytes in the liver is low, ranging from 5% to 20% [8], [9], [11], [39], supporting a model of genetic compartmentalization. Wieland and colleagues have suggested that infected foci comprising <5% of hepatocytes in the liver occur when the plasma HCV RNA burden is <10^5^ copies/ml and that above this threshold the frequency of infected hepatocytes can range from 20% to 50%. In our cohort 18 of the 21 patients showed plasma HCV RNA levels well above this threshold (Fig. 2), suggesting that ‘local’ compartmentalization within a segment may be lost in late stage disease when the frequency of infected cells is high. In contrast to the limited intra-hepatic variation in HCV RNA levels we noted significant differences between patients of up to 500-fold [11], [39]. These differences were unrelated to the patient’s age, indication for transplantation, or infecting HCV genotype (Table 1). Although it is possible that patients with high hepatic viral loads will have more HCV infected cells or higher amounts of HCV RNA per infected cell, the complex microscopic approaches to visualize these cells using HCV core or NS5A antibodies or patient-specific viral nucleic acids were beyond the scope of this study [8], [11].

One could predict that the diversity of HCV quasispecies would decrease in late stage disease due to a reduced immune response, while the more active immune responses in the earlier phases of infection may select for a greater diversity of HCV quasispecies [44], [45]. HCV sequence variation was readily detectable, though it is not possible to distinguish whether this is in response to ongoing selection or a relic of earlier evolutionary factors. We assessed genetic polymorphism using both single gene amplification and deep sequencing approaches. Although SGA cloning and sequencing methods offer the advantage of identifying whole regions of the virus, it is costly and the number of clones that can be obtained is limited [46], [47], [48], [49]. Deep sequencing approaches overcome the relatively poor sampling inherent in SGA by analyzing thousands of short viral gene fragments. This allows us to base our calculations on many hundreds of sequences and derive our diversity estimates with complete precision. In this study, the average number of reads for each base of the E1E2 region was 3045–11,824, which is much larger than previous studies often with <20 sequences reported [46], [47], [48], [49]. Interestingly we were able to demonstrate that, even with the limited sampling inherent in the method, our SGA-based inferences of genetic diversity closely reflected those obtained from deep sequencing. Furthermore, the sequences of our SGA clones were the most abundant members of the quasispecies identified by deep sequencing (Table 2), demonstrating that SGA recovers the most representative sequences in the population.

Comparing liver-derived HCV quasispecies with plasma showed a high degree of overlap, though the relative abundance of the variants differed between the two compartments and a few unique sequences were observed in each sample (Table 3). These differences may reflect the hepatic retention of poorly released (cell-to-cell transmitted) HCV variants [13]. Alternatively neutralizing antibodies may mediate the clearance of some viruses, leading to an over representation of neutralization escape mutants in the periphery [3], [50], [51]. Extra-hepatic viral reservoirs are another potential source of viruses in the plasma that were not detected in the liver, a model supported by the detection of HCV negative strand RNA in PBMCs [52], [53], lymph nodes [54] and the central nervous system [55]. These extra-hepatic sites have been suggested to serve as reservoirs for viruses to re-infect the allograft after transplantation [56], [57], [58], [59], [60], [61].

To the best of our knowledge these data represent the most comprehensive study on intra-hepatic HCV compartmentalization to date using both structural and non-structural proteins. It reveals high levels of sequence similarities between different sites in the liver, supported by all HVR1 sequences being present in at least 2 hepatic segments and by sub-populations of dominant sequences being identical across segments. Several studies have compared HCV sequences in the liver and plasma. Some report a genetic relationship between viral strains present in both compartments [3], [42] with half of the patients sharing the same consensus sequence in plasma and liver, together with a unique site-specific set of minor sequences [3], [42], [50]. In contrast, Fan and colleagues found no evidence for association between these sites [51]. These high genetic similarities between the hepatic segments may reflect a long history of chronic infection where genetically distinct clusters have been lost over time.

Sobesky and colleagues [62] reported HCV compartmentalization between tumor and non-tumor regions of the liver in 5 of 7 patients diagnosed with HCC. Consistent with these findings a recent study by Harouaka *et al.* reported differences in HCV quasispecies distribution between tumor and non-tumor compartments [63], supporting a model where HCV may evolve differently in non-tumor cirrhotic nodules. However, both of these studies assessed genetic compartmentalization using the matrix-based Mantel’s test of genetic and anatomical distances that has been criticized for its statistical performance [64], [65]. We therefore used the more rigorous SM test to evaluate compartmentalization, which infers the number of migration events between compartments using the structure of the reconstructed phylogenetic tree. While two subjects (patients 4 and 7) were diagnosed with HCC, no evidence for E1E2 compartmentalization was obtained. HCC was not detected in all 8 segments of these livers, therefore our comparisons focused on non-HCC biopsies. Many reports of HCV quasispecies genesis are based on a demic model of viral evolution where isolated variants arise in geographically distinct locations. However, our data on HCV quasispecies in the explanted liver and plasma suggest that in late stage liver disease HCV compartmentalization does not occur.

## Financial support

Our research is supported by: The Birmingham Children’s Hospital Research Foundation, the National Institute for Health Research Birmingham Liver Biomedical Research Unit (JM); MRC grant G1100247 (JM & PB); EU FP7 funded PathCO HEALTH F3-2012–305578 (JM); the National Institute of Allergy and Infectious Diseases under grant U19-AI082630 (TM).

## Conflict of interest

The authors who have taken part in this study declare that they do not have anything to disclose regarding funding or conflict of interest with respect to this manuscript.

## Authors’ contributions

DLH, DCT, IAH, GMR, DJB, KH, CD and PB performed the work described here. AW, CBO, KAP, AT and DK provided key expertise and reagents. DLH, DK, TMA, JAM and PB designed the study. DLH, DCT, JAM and PB wrote the manuscript.
